# Eczema of the Lips Caused by Mouth Washes and Tooth Powders

**Published:** 1899-10

**Authors:** 


### Eczema of the Lips Caused by Mouth Washes and Tooth Powders,

Was observed by Prof. Weisser (Breslau) in a few cases. A boy, six years of age, was suffering for months from a squamous eczema, surrounding the mouth. All treatment employed proved unsuccessful; but as soon as the mouth wash was discontinued the eczema at once disappeared. A similar experience was had in a second and a third case. In a fourth, which occurred in a young lady, the affection had existed for two years, obliging the patient to continuously apply ointments. After all mouth washes and tooth powders which contained olive oil or peppermint oil were relinquished, a marked improvement of the eczema occurred. According to this, we are seemingly justified in ascribing to the ethereal oils, usually present in mouth washes and tooth powders, a bad effect on dermal affections occurring in the vicinity of the lips and oral cavity.—*Wien. Klin. Wochenschr.*

